# Endogenous female sex hormones delay the development of renal dysfunction in apolipoprotein E-deficient mice

**DOI:** 10.1186/1476-511X-13-176

**Published:** 2014-11-25

**Authors:** Sonila S Carneiro, Raffaela Z Carminati, Flavia PS Freitas, Priscila L Podratz, Camille M Balarini, Jones B Graceli, Silvana S Meyrelles, Elisardo C Vasquez, Agata L Gava

**Affiliations:** Laboratory of Translational Physiology, Physiological Sciences Graduate Program, Health Sciences Center, Federal University of Espirito Santo, Av Marechal Campos 1468, 29042-755 Vitoria, ES Brazil; Morphology Department, Health Sciences Center, Federal University of Espirito Santo, Vitoria, ES Brazil; Department of Physiology and Pathology, Health Sciences Center, Federal University of Paraiba, Joao Pessoa, PB Brazil; Division of Pediatric Endocrinology, John Hopkins University, School of Medicine, 600 N. Wolfe Street/Room 4-106, Baltimore, MD 21287 USA; Pharmaceutical Sciences Graduate Program, University of Vila Velha, Vila Velha, ES Brazil; Laboratory of Cellular Toxicology, Physiological Sciences Graduate Program, Health Sciences Center, Federal University of Espirito Santo, Av Marechal Campos 1468, 29042-755 Vitoria, ES Brazil

**Keywords:** Female sex hormones, Hypercholesterolemia, Renal function

## Abstract

**Background:**

Hypercholesterolemia is a well-established risk factor for the development of kidney injury. Considering that female sex hormones may play a preventative role in both cardiovascular and renal diseases, the aim of the present study was to evaluate the effects of female sex hormones on hypercholesterolemia-induced renal dysfunction.

**Methods:**

Apolipoprotein E-deficient (ApoE) and C57 control female mice underwent an ovariectomy (OVX) or sham surgery and after 2 months, creatinine clearance, uremia and proteinuria were determined. Renal oxidative stress and lipid deposition were also quantified. Values are presented as mean ± SEM. Statistical analyses were performed using Two-way ANOVA followed by Tukey’s post hoc test.

**Results:**

Creatinine clearance (μL/min) was similar between C57 (171 ± 17) and ApoE (140 ± 26) mice underwent sham surgery. OVX resulted in a reduced glomerular filtration rate in both C57 (112 ± 8, ~ − 35%, p < 0.05) and ApoE (61 ± 10, ~ − 56%, p < 0.05) animals. Plasma levels of urea (mg/dL) were higher in both ApoE groups (Sham: 73 ± 7; OVX: 73 ± 8, p < 0.05) when compared to C57 animals (Sham: 49 ± 3; OVX: 60 ± 4), with no changes among ovariectomized groups. Proteinuria levels (mg/24 h) were similar between C57 (Sham: 25.1 ± 5.7; OVX: 33.7 ± 4.7) and ApoE sham animals (26.4 ± 3.5), however, 24-h urine protein excretion was augmented in ApoE OVX animals (49.6 ± 5.8, p < 0.05). Histological kidney analysis demonstrated that the absence of female sex hormones resulted in increased oxidative stress, which was more severe in ApoE mice (C57 Sham: 9.2 ± 0.4; C57 OVX: 22.9 ± 1.0; ApoE Sham: 13.9 ± 0.7; ApoE OVX: 34.0 ± 1.4 au x 10^3^, p < 0.05). As expected, ApoE mice presented higher lipid deposition, which was not affected by OVX (C57 Sham: 0 ± 0; C57 OVX: 0 ± 0; ApoE Sham: 6.8 ± 1.6; ApoE OVX: 5.2 ± 0.8% x 10^−2^, p < 0.05). Ovariectomy resulted in a similar reduction in ER-α protein expression in the renal cortex (C57: 0.78 ± 0.04; ApoE: 0.81 ± 0.04 au, p < 0.05) when compared to sham animals (C57:1.00 ± 0.04; ApoE: 1.03 ± 0.03 au).

**Conclusion:**

Taken together these data indicate that female sex hormones may delay hypercholesterolemia-induced renal dysfunction and emphasizes the importance of plasma cholesterol control in post-menopausal women.

## Background

Atherosclerosis is a complex disorder that leads to premature death and hospitalization [1]. Vascular lipid deposition and the resultant cardiovascular complications, such as myocardial infarction, stroke and ischemic heart failure, are the major cause of death in the Western world [2]. Although cardiovascular outcomes can be considered one of the most significant consequences of atherosclerosis, this disease can also contribute to the development and/or progression of renal diseases, such as chronic kidney disease (CKD) [3].

Experimentally, atherosclerosis can be induced by the inactivation of the apolipoprotein-E (apoE) gene by homologous recombination [4, 5]. Among the genetically engineered models, the apoE-deficient (apoE^−^/^−^) mouse is considered to be one of the most relevant models because it develops spontaneous hypercholesterolemia and arterial lesions similar to those observed in humans [6]. ApoE^−/−^ mice present higher levels of plasma cholesterol when compared to wild-type animals. The lipid profile in mice differs from that in humans, who carry most of the serum cholesterol in LDL particles. Normally, mice do not express cholesterol-ester transfer protein (CETP) and thus carry most of their plasma cholesterol in HDL particles [7]. However apoE^−/−^ mice lipid profile resembles that found in humans, once they present a shift in plasma lipoprotein from HDL to cholesterol-rich remnants of chylomicrons and VLDL [8].

The main risk factors associated with atherosclerosis are well known, including hypertension, diabetes, smoking and increased serum total and low-density lipoprotein cholesterol [2]. Among these, it is interesting to note that hypercholesterolemia is also considered a contributing factor towards the development of renal dysfunction [9]. In fact, the prevalence of dyslipidemia in patients with CKD is much greater than in the general population [10]. On the other hand, the progressive deterioration of renal function associated with CKD may also lead to dyslipidemia, which contributes to the development of atherosclerosis [11]. As such, dyslipidemia and atherosclerosis accelerate renal dysfunction, which in turn, promotes atherosclerosis.

Gender is a non-modifiable risk factor that influences the progression of atherosclerosis and renal diseases. The majority of studies show that during their reproductive years, women are less prone to developing cardiovascular disease and atherosclerosis, but men and post-menopausal women of comparable age are at equal risk [12]. Furthermore, population-based studies indicate that men present a more severe CKD progression than women [13, 14]. Estrogens can mediate several beneficial effects on renal function, including suppression of extracellular matrix synthesis in the glomerular mesangial cells [15], reduced tubulointersticial fibrosis [16] and increased NO production and/or bioavailability [17, 18]. Based on these data, the goal of the present study was to evaluate the protective role of endogenous female sex hormones on hypercholesterolemia-induced renal dysfunction in apoliprotein-E deficient mice, an experimental murine model of hypercholesterolemia and atherosclerosis. Our data demonstrate that the ovariectomy led to a significant loss of renal function in hypercholesterolemic mice.

## Results

The results of body weight, dry uterus weight and the uterus atrophy index, given by the ratio between dry uterus weight and body weight, are demonstrated in Table 1. Body weight was similar between all groups. As expected, dry uterus weight was smaller in OVX animals when compared to control groups, with no difference between C57 and ApoE animals. As consequence, dry uterus weight/body ratio was also diminished in ovariectomized animals (approximately 84%) when compared to sham groups, confirming the efficiency of the OVX procedure.

**Figure 1:**
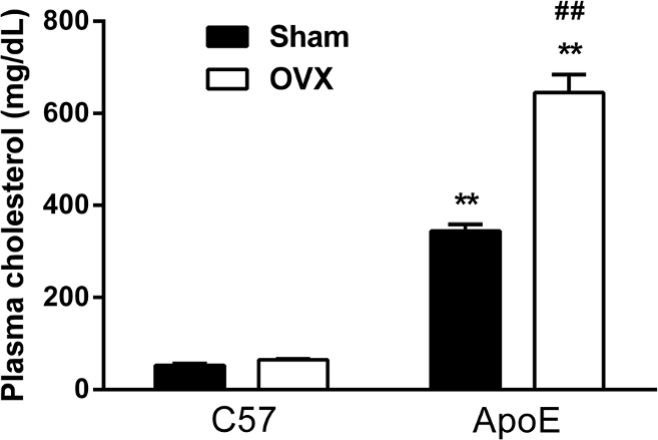
**Evaluation of the effects of endogenous female sex hormones removal on total plasma cholesterol levels.** As expected, ApoE mice present hypercholesterolemia, which was further increased after ovariectomy. C57 Sham n = 7, C57 OVX n = 6, ApoE Sham n = 7, ApoE OVX n = 9. Values are means ± SEMs. **p < 0.01 vs. C57; ^##^p < 0.01 vs. respective control. Two-way ANOVA followed by Tukey’s post hoc test.

The effects of endogenous female sex hormones removal on plasma cholesterol levels are displayed in Figure 1. As expected, ApoE mice present hypercholesterolemia (Sham: 345 ± 14 mg/dL, p < 0.01) when compared to C57 animals (Sham: 52 ± 4 mg/dL). Ovariectomy did not modify plasma cholesterol levels in the C57 OVX group (64 ± 2 mg/dL), however it intensified hypercholesterolemia in the ApoE OVX group (645 ± 38 mg/dL, p < 0.01). These data indicate that endogenous female sex hormones can have a protective role against hypercholesterolemia.

**Figure 2:**
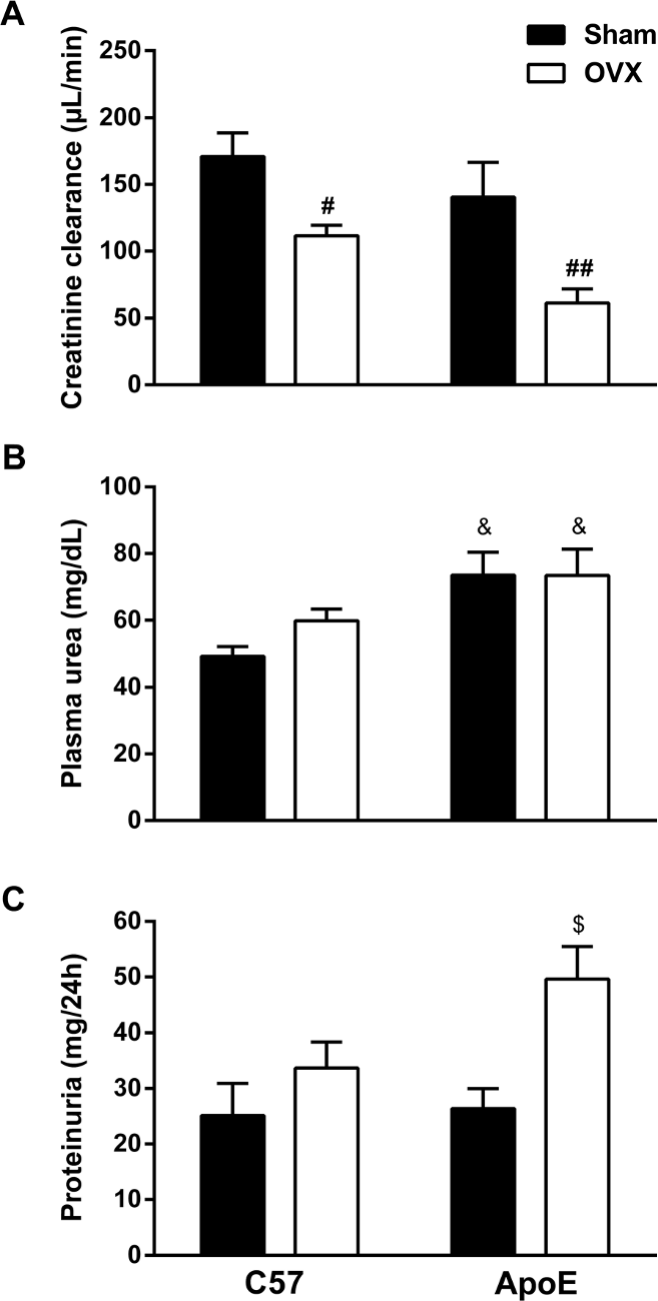
**Renal function evaluation using creatinine clearance (A) plasma levels of urea (B) and proteinuria (C).** The association of hypercholesterolemia and ovariectomy resulted in a marked renal dysfunction, as demonstrated by the reduced glomerular filtration rate and increased uremia and proteinuria. C57 Sham n = 10-12, C57 OVX n = 8-11, ApoE Sham n = 7-13, ApoE OVX n = 7-12. Values are means ± SEMs. ^#^p < 0.05 and ^##^p < 0.01 vs. respective control; &p < 0.05 vs. C57 sham; ^$^p < 0.05 vs. all other groups. Two-way ANOVA followed by Tukey’s post hoc test.

Renal function was determined using creatinine clearance, plasma values of urea and urine protein excretion in all studied groups; these results are demonstrated in Figure 2. Hypercholesterolemia did not cause any significant changes in the glomerular filtration rate of female ApoE mice (C57 Sham: 171 ± 17; ApoE Sham: 140 ± 26 μL/min). The removal of endogenous female sex hormones resulted in decreased creatinine clearance in both C57 OVX (112 ± 8 μL/min, p < 0.05) and ApoE OVX (61 ± 10 μL/min, p < 0.01) groups; nevertheless, this reduction was greater in the ApoE OVX group (approximately 56%). Plasma values of urea (Figure 2B) were increased in ApoE animals (Sham: 73 ± 7; OVX: 73 ± 8 mg/dL, p < 0.05) when compared to C57 group (Sham: 49 ± 3 mg/dL). However, ovariectomy did not modify uremia in either C57 OVX (60 ± 4 mg/dL) or ApoE OVX when compared to their respective controls. Evaluation of the 24-hour urine protein excretion (Figure 2B) revealed that neither hypercholesterolemia (ApoE Sham: 26.4 ± 3.5 mg/24 h) nor ovariectomy (C57 OVX: 33.7 ± 4.7 mg/24 h) resulted in altered proteinuria when compared to C57 Sham (25.1 ± 5.7 mg/24 h). However, when these two situations were concomitant, 24-hour urine protein excretion is augmented (ApoE OVX: 49.6 ± 5.8 mg/24 h, p < 0.01). Taken together, these data indicate that endogenous female sex hormones can delay hypercholesterolemia-induced renal dysfunction.

**Figure 3:**
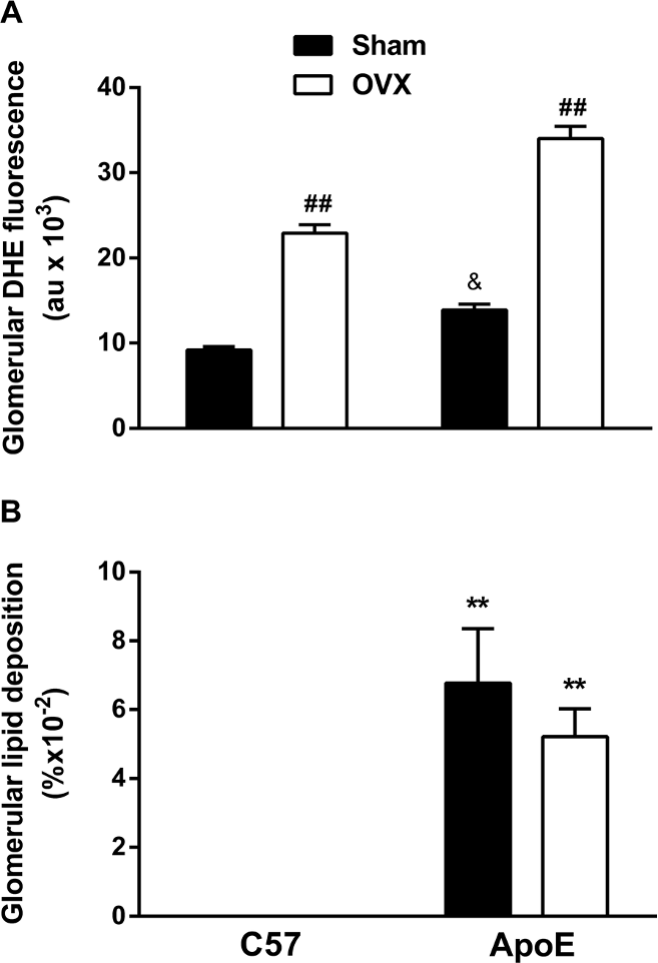
**Glomerular histological quantification of superoxide anion production (A) and lipid deposition (B). (A)** Hypercholesterolemia resulted in elevated glomerular oxidative stress, which was exacerbated by the removal of endogenous female sex hormones. **(B)** As expected, lipid deposition was enhanced in ApoE groups, but it was not influenced by ovariectomy. C57 n = 4-7, ApoE n = 7-9. Values are means ± SEMs. ^##^p < 0.01 vs. respective control; &p < 0.05 vs. C57 sham; **p < 0.01 vs. C57. Two-way ANOVA followed by Tukey’s post hoc test.

The histological evaluation for glomerular superoxide anion production (DHE fluorescence – A) and glomerular lipid deposition (Oil-Red-O staining – B) were performed in C57 and ApoE mice (Figure 3). As expected, ApoE animals presented increased oxidative stress into the glomerulus (ApoE Sham: 13.0 ± 0.7 au, p < 0.05) when compared to the C57 group (C57 Sham: 9.2 ± 0.4 au). The removal of endogenous female sex hormones resulted in augmented superoxide anion production in both C57 (C57 OVX: 22.8 ± 1.0 au) and ApoE (34.0 ± 1.4 au) mice (Figure 3A). The quantification of glomerular Oil-Red-O staining (Figure 3B) demonstrated no lipid deposition in either C57 Sham (0 ± 0%) or C57 OVX (0 ± 0%). ApoE mice exhibited increased glomerular lipid deposition (ApoE Sham: 0.07 ± 0.01%), which was not exacerbated by ovariectomy (ApoE OVX: 0.052 ± 0.01%).

**Figure 4:**
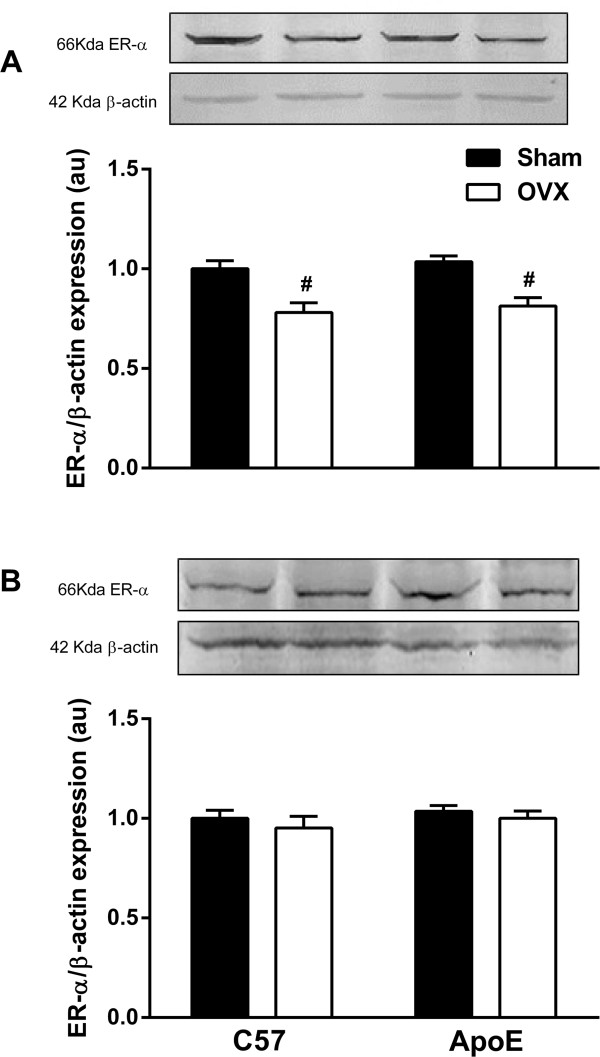
**Western blot analysis of ER-α expression in the renal cortex (A) and medulla (B). (A)**: The removal of endogenous female sex hormones resulted in reduced ER-α expression in renal cortex in both C57 and ApoE groups. **(B)** Neither hypercholesterolemia nor ovariectomy affected medullary ER-α expression. All groups have n = 4. Values are means ± SEMs. ^#^p < 0.05 vs. respective control. Two-way ANOVA followed by Tukey’s post hoc test.

ER-α protein expression was evaluated in both the renal cortex and medulla from C57 and ApoE mice via Western blot analysis (Figure 4). Immunoblotting of the renal cortex (A) demonstrated that ERα protein expression was reduced by ovariectomy in C57 (OVX: 0.78 ± 0.05 au) and ApoE (OVX: 0.81 ± 0.04 au) animals when compared to their respective controls (C57 Sham: 1.00 ± 0.04; ApoE Sham: 1.04 ± 0.03 au). Medullary ERα protein expression (B) was affected by neither hypercholesterolemia nor ovariectomy (C57 Sham: 1.00 ± 0.04; C57 OVX: 1.03 ± 0.02; ApoE Sham: 0.95 ± 0.06; ApoE OVX: 1.00 ± 0.03 au).

**Table 1:** **Body weight, dry uterus weight and uterus atrophy index in all studied groups**

Parameters	Groups			
	C57 Sham (8)	C57 OVX (5)	ApoE Sham (10)	ApoE OVX (6)
Body weight (g)	21.8 ± 0.7	21.9 ± 0.7	22.0 ± 0.9	22.2 ± 0.9
Uterus weight (mg)	13.5 ± 1.6	2.1 ± 0.1*	15.4 ± 1.1	2.5 ± 0.5*
Uterus atrophy index (mg/g)	0.62 ± 0.07	0.10 ± 0.01*	0.7 ± 0.01	0.11 ± 0.02*

All values are expressed as means ± SEMs. The number in parentheses represents the number of animals in each group. *p < 0.05 vs. sham animals.

## Discussion

In the present study, we evaluated the effects of endogenous female sex hormones on the renal function of hypercholesterolemic mice. Our data demonstrates that the removal of the ovaries from ApoE mice lead to remarkable renal dysfunction, indicating that endogenous female sex hormones can play a protective role against hypercholesterolemia-induced kidney injury.

Female endogenous sex hormones, especially the steroid hormone 17β-estradiol plays critical roles in the reproductive tract, mammary gland, brain, skeletal and cardiovascular systems [19, 20]. In the reproductive tract, the role of estrogen in uterine epithelial cell division and cell death has been extensively studied, and the results demonstrate that estrogen induces cell division and prevents cell death in this tissue [21]. As expected, the present investigation revealed that mice underwent ovariectomy exhibited remarkable uterine atrophy when compared to control animals, with no differences between C57 and ApoE mice. Previous studies from our laboratory also demonstrated that the removal of endogenous female hormones results in a decreased uterus weight similar to C57 and ApoE mice [22]. Although we did not measure plasma levels of female hormones, the reduction in uterus weight can be considered a good marker of the efficacy of ovariectomy surgery, as previously reported [22, 23].

In the present investigation, plasma cholesterol levels were elevated in ApoE mice compared to C57 controls. Ovariectomy did not modify this parameter in C57 animals; however, it exacerbated hypercholesterolemia in the ApoE OVX group. It is well established that estrogens are important modulators of lipid metabolism and that premenopausal women are largely protected from a variety of cardiovascular diseases, including dyslipidemia [24]. High serum estrogen levels are associated with favorable lipid profiles, as evidenced by increased HDL-cholesterol and decreased LDL-cholesterol levels [24]. Estrogens induce these alterations through a variety of mechanisms, such as reduction of LDL oxidation [25], changes in LDL receptor expression [26] and decreased hepatic lipase dependent catabolism of HDL particles [27].

Renal function was evaluated using creatinine clearance, plasma levels of urea and daily protein excretion. The removal of endogenous female hormones resulted in a slight reduction in the renal function of C57 animals; however, this effect was much more prominent in ApoE animals. In contrasting with previous data from our laboratory showing that young male ApoE mice present a marked renal dysfunction [28], the present study indicates that female ApoE mice are protected from renal injury, as they present a normal creatinine clearance. Despite this observation, ApoE mice also have greater levels of uremia and increased renal oxidative stress, indicating that although the glomerular filtration rate was not significantly altered, these animals may already present a reduced renal reserve. As a consequence, when the kidney is challenged by other risk factors, such as the reduction in female sex hormones, the loss of renal function is intensified, as we observed in the ApoE OVX group.

Although several studies have investigated the protective effects of female hormones on the kidney, the underlying mechanisms remain to be elucidated. Estrogens are able to suppress extracellular matrix synthesis in glomerular mesangial cells through MAPK activity modulation [15] and by modulating transforming growth factor-β protein expression and signaling [29]. Furthermore, estrogens reduce tubulointersticial fibrosis through increased matrix metalloproteinase activity [16], which limits the progression of glomerulosclerosis. In addition, estrogens can also contribute to podocyte actin stabilization and protect podocytes against oxidant-induced injury, as demonstrated by Catanuto et al. [30, 31]. Taken together, these data indicate that female sex hormones can play a protective role in the development and progression of glomerulosclerosis, and consequently prevent or delay the loss of renal function.

Another important effect of estrogen relies on renal nitric oxide (NO) synthesis and/or bioavailability. The NO system consists of three distinct NO synthase (NOS) isoforms, encoded by three distinct genes, including neuronal (nNOS or NOS-1), inducible (iNOS or NOS-2) and endothelial (eNOS or NOS-3) [32]. All of the NO isoforms have been identified in the kidney [33]. NO plays numerous physiological roles in the kidney, including control of renal and glomerular hemodynamics, through interference at multiple physiologically critical steps of nephron function [34]. Estrogens may upregulate eNOS mRNA in renal medullary cells, increase NOS activity and upregulate eNOS protein levels [17]. Corroborating these data, Pérez-Torres [35] demonstrated that in ovariectomized female rats with metabolic syndrome, eNOS expression was significantly lower than in intact animals, indicating that female sex hormones can modulate the renal synthesis of NO. However, changes in NOS expression do not always correlate with NO bioavailability, superoxide anions react extremely rapidly with NO, generating peroxynitrite. Under physiological conditions, this interaction is minimized by endogenous antioxidant defenses, such as superoxide dismutase activity [36]. Nevertheless, during conditions of increased oxidative stress, such as hypercholesterolemia [37], these defenses may not be able to compensate and protect the cells against reactive oxygen species (ROS)-induced damage. In our study, ApoE mice present higher levels of DHE fluorescence in the glomerulus, indicating an increased superoxide anion generation. However, in both ovariectomized groups, especially in ApoE OVX animals, glomerular oxidative stress was further augmented, indicating a protective role for endogenous female sex hormones on ROS generation. Corroborating our data, Borras et al. [18] reported that females have greater concentrations of antioxidant enzymes, resulting in lower production of ROS and that ovariectomy increases ROS generation. Strehlow et al. [38] reported that 17-β estradiol upregulates MnSOD and extracellular SOD expression and activity. Therefore, it seems that increased oxidative stress may be a potential mechanism by which the removal of endogenous female sex hormones resulted in renal dysfunction in ApoE mice.

Because ApoE OVX animals presented greater levels of plasma cholesterol than their respective controls, we sought to investigate if the observed increase in hypercholesterolemia contributed to the loss of renal function due to augmented lipid deposition in the kidney. However, we observed similar lipid deposition in both ApoE groups, indicating that this alteration is not likely to be involved in the exacerbated renal dysfunction found in ApoE ovariectomized animals. In contrast with our data, several studies have demonstrated a beneficial effect of estrogen on the formation of atherosclerotic lesions, including inhibition of the production and activity of growth factors [39], reduced inflammation [40], improvement of endothelial function [41], and an ameliorated lipid profile [42]. However, the majority of studies suggest that the protective effects of estrogen in animal models of atherosclerosis are mediated by ER-α [43]. In our study, the protein expression of ER-α was diminished in the renal cortex, which might have contributed to similar levels of glomerular lipid deposition in both ovariectomized and sham ApoE animals.

We also investigated the effects of hypercholesterolemia and endogenous female sex hormones removal on estrogen receptor (ER) expression. Under physiological conditions, the biological effects of estrogen depend not only the level of estrogen but also on the distribution and expression levels of the corresponding ERs, ER-α and ER-β in the target cell [43]. The kidney contains many ERs and numerous estrogen-regulated genes, which are primarily regulated by ER-α [44]. In our study, both OVX groups presented a reduced ER-α protein expression in the renal cortex, with no alterations in the renal medulla. In accordance with our data, Esqueda et al. [45] also demonstrated a diminished ER-α expression in the renal cortex following ovariectomy in Dahl salt-sensitive rats. This effect is also observed in other tissues, such as the uterus and vagina [43]. Interestingly, in other models of renal injury, such as diabetic nephropathy, there is an association between decreased circulating estrogen levels and reduced renal ER protein expression [46, 47]. We propose that the decrease in ER-α expression in mice after ovariectomy contributes to the development of renal damage, especially in the ApoE OVX group.

Although the most prominent effects of ovariectomy are attributed to the lack of estrogen, we cannot rule out the consequences of progesterone deficiency. In general, endogenous progesterone augments the beneficial effects of estrogen [48]. Progesterone can increase endothelium-dependent relaxation [49] and inhibit mitogen-induced growth and proliferation of mesangial cells [50]. Additionally, progesterone can modulate renal function through changes in sympathetic nervous system, decreased cathecholamine secretion by the adrenal medulla [51] and reduced gamma-amminobutyric acid inhibitory effects on rostral ventrolateral medulla [52]. However, more studies are necessary to elucidate the effects of progesterone on the kidney in both normal and pathophysiological situations.

## Conclusion

Endogenous female sex hormones removal led to renal dysfunction, which was remarkably increased in ApoE animals. The mechanisms involved in this renal injury may include augmented oxidative stress and reduced ERα expression. This investigation highlights the significance of endogenous female sex in maintaining renal homeostasis, especially when associated with other risk factors, such as hypercholesterolemia and emphasizes the importance of plasma cholesterol control in post-menopausal women.

## Methods

### Animals and experimental protocol

The experiments were performed using female C57BL/6 (C57) and ApoE knockout mice (ApoE). The apoE gene was inactivated by homologous recombination in mouse embryonic stem cells, usually in a C57 genetic background. The animals were obtained from the animal facilities of the Health Sciences Center at the Federal University of Espirito Santo, housed according to institutional guidelines for animal research and fed a normal diet. The procedures were previously approved by the institutional Ethics Committee for Use of Animals from the Research Center of Emescam College of Health Sciences (CEUA-EMESCAM, protocol #006/2010) and were conducted in accordance with the international guidelines for care and use of laboratory animals. All surgery was performed under sodium ketamine + xylazine anesthesia (91.0/9.1 mg/kg, *i.p.*), and all efforts were made to minimize suffering.

At 4 weeks of age, C57 and ApoE mice underwent ovariectomy or sham surgery as previously described [53]. Briefly, after an abdominal incision, the ovaries were clamped and removed, and the skin was then sutured before the animals were returned to their cages. The efficacy of the ovariectomy was confirmed two months after surgery, using uterus weight/body weight ratio and by the evaluation of the estrous cycle. As previously described [54], in wild-type mouse the major population of cells in proestrous are nucleated cells; estrous, cornified; metaestrous, leukocytes; diestrous leukocytes. The animals were considered ovariectomized when the microscopic evaluation of vaginal smears showed lack of cells or very few cornified cells. Sham animals were used to experiments during estrous phase of cycle.

After the vaginal smears evaluation, the animals were housed individually in metabolic cages to allow 24-hour urine volume determination and urine collection. Then, the animals were euthanized with an overdose of thiopental (Cristalia, Sao Paulo, Brazil, 200 mg/kg, i.p.) and blood was collected for creatinine, cholesterol and urea measurements. Tissues were perfused with cold phosphate- buffered saline (PBS, pH 7.4, 0.1 M) through the left ventricle and one kidney was removed for Western blot analysis. The remaining kidney was fixed in paraformaldehyde 4% in PBS for histological evaluation. The uterus was also removed, dried at 37°C for 24 hours and weighed to confirm the effectiveness of ovariectomy surgery. Serum cholesterol, urea, creatinine and urine creatinine were measured using colorimetric kits (Bioclin®, Belo Horizonte, Brazil). Proteinuria was determined in the urine samples by the Bradford method [55]. Creatinine clearance was calculated using plasma and urine creatinine concentrations and urine flow using the standardized formula.

### Renal histological analysis

After perfusion, the kidneys were embedded in OCT compound (Tissue-Tek; Sakura Finetek USA, Torrance, CA, USA) and stored at −80°C until further use. Eight μm cryosections (Leica, CM 1850, Leica, Wetzlar, Germany) were prepared and mounted on a parallel series of gelatin coated slides and stained with Oil-Red-O (Sigma- Aldrich) to detect neutral lipids or labeled with the fluorescent dye dihydroethidium (2 μM) to observe reactive oxygen species formation as previously described [56]. To verify lipid deposition, the images were captured with a color video camera (VKC150; Hitachi, Tokyo, Japan) connected to a microscope (AX70; Olympus, Center Valley, PA) and analyzed with Image J software (National Institute of Health). To determine oxidative stress, the tissue sections were imaged using a fluorescence microscope (Nikon Ti-S) with a G-2E/C Nikon filter that provided excitation at 530 nm and emission at 610 nm. All quantifications were performed by a single blind subject.

### Protein extraction and Western blotting

The renal cortex and medulla were dissected using a Sttadie-Rigg microtome and used as separate tissue pools; respective tissues were homogenized as previously described [57]. Proteins were solubilized by heating at 100°C for 1 min in sample buffer (62.5 mM Tris–HCl, pH 6.8, 2% sodium dodecyl sulfate (SDS), 5% glycerol, 0.01% bromophenol blue, and 1.7% β-mercaptoethanol). Standard SDS-polyacrylamide gel electrophoresis (PAGE) was carried out by loading equal quantities of protein per lane (100 μg) into a 10% SDS-polyacrylamide gel. Proteins were transferred onto nitrocellulose membranes (BioRad, Hercules, CA) in Tris–glycine transfer buffer and incubated with a rabbit polyclonal antibody raised against the C-terminus of estrogen receptor alpha (ER-α, 1:500 in 3% nonfat dried milk in TBST, overnight, at 4°C, Santa Cruz, INC) or rabbit polyclonal antibody raised against the C-terminus of βactin (1:1000 in 3% nonfat dried milk in TBST, overnight, at 4°C, Santa Cruz, INC). Goat anti-rabbit IgG conjugated with alkaline phosphatase (Sigma Immuno-Chemicals) was used as a secondary antibody (ER-α: 1:1000; β-actin: 1:4000, respectively). The blots for ER-α and β-actin (used as an internal control) were visualized by a color development reaction using nitroblue tetrazolium chloride (NBT) and 50 mg/mL of 5-bromo-4-chloro-3-indolylphosphate p-toluidine salt (BCIP) (all from Life Technologies, Rockville, MD) for 5 min. The ERα and βactin bands were analyzed by densitometry using Image J software. Relative expression was normalized by dividing the ER-α values by the corresponding internal control values (β-actin).

### Statistical analyses

All data are expressed as the mean ± SEM. Statistical analysis was performed using two-way ANOVA, followed by Tukey’s post hoc test. The level of significance was set at p < 0.05.
